# **Genetic analysis and phytochemical profile of soursop (*Annona muricata* L**.**) cultivated in family orchards in southeastern Mexico**

**DOI:** 10.1371/journal.pone.0321846

**Published:** 2025-05-07

**Authors:** Heidi Beatriz Montejo-Mendez, Julia Maria Lesher-Gordillo, Jose I. Hormaza, Carlos Ernesto Lobato-Garcia, Abraham Gomez-Rivera, Salima Machkour-M’Rabet, Manuel Ignacio Gallardo-Alvarez, Nerea Larranaga, Aminta Hernandez-Marin, Alejandra Valdes-Marin, Ricardo Lopez-Rodriguez, Yann Henaut, Hilda María Díaz-Lopez

**Affiliations:** 1 División Académica de Ciencias Biológicas, Universidad Juárez Autónoma de Tabasco, Villahermosa, Tabasco, Mexico; 2 Instituto de Hortofruticultura Subtropical y Mediterránea La Mayora (IHSM La Mayora – CSIC - UMA), Algarrobo-Costa, Málaga; 3 División Académica de Ciencias Básicas, Universidad Juárez Autonoma de Tabasco, Cunduacán, Tabasco, Mexico; 4 Departamento de Conservación de la Biodiversidad, El Colegio de la Frontera Sur, Unidad Chetumal, Quintana Roo, Mexico; West Bengal University of Animal and Fishery Sciences, INDIA

## Abstract

*Annona muricata* is an important and widespread neotropical perennial fruit tree, that has received increasing attention in recent years for its pharmaceutical potential, particularly for the presence of metabolites with reported anti-tumoral properties. In this study, 191 samples of this species were collected from homegardens across four states of southern Mexico. and analyzed using a dual approach, including genetic and chemical analyses. The local genetic diversity and population structure was determined through the analysis of 10 microsatellite loci- The metabolic content of flavonoids and polyphenols was analyzed at ten localities. Genetic diversity was found to be low to moderate in all populations with values of expected heterozygosity (*H*_*e*)_ ranging from 0.28 to 0.52. Our research indicated the presence of three distinct genetic groups, which did not appear to be associated with geographical origin. Variations in the chemical content of flavonoids and polyphenols were observed among the different locations examined, with flavonoid values ranging between 73.48 and 592.70 mg RE/gE and polyphenol values ranging from 13.10 to 126.59 mg GAE/gE. Accessions from Champoton and Emiliano Zapata demonstrated the highest flavonoid content, while Palenque, Champoton and Centro presented the highest polyphenol content. Multidimensional scaling (MDS) analysis revealed a correlation between genetic profiles, chemical profiles and the influence of human activity in the populations examined. This analysis revealed significant genetic differences between these populations, suggesting that they are associated with different levels of chemical contents. Remarkably, highly disturbed sites appeared to correlate with a considerable increase in chemical content.

## Introduction

*Annona muricata L.,* commonly known as guanabana or graviola, is a species of great economic importance within the *Annonaceae* family, widely cultivated in tropical regions of Central and South America, West, Central and East Africa, and Southeast Asia [1]. The main producing countries include Brazil, Mexico and Venezuela [2]. In Mexico, soursop cultivation covers an area of 2,702 ha, according to SIAP reports (2018), with three registered national clonal varieties Guanay-1, Guanay-2 and Guanay-3.

The *Annonaceae*, to which soursop belongs, comprises approximately 107 genera and 2,400 species worldwide [3]. In accordance with the APGIV classification system [4], this family is situated within the order Magnoliales, which forms part of the Magnoliid complex. This complex also encompasses other orders, including the Canellales, Laurales and Piperales, which are regarded as sister groups of the eudicotyledons and monocotyledons. [5]. In tropical climates, soursop produces fruit throughout the year, with peak production in spring and early autumn. Its propagation is mainly by grafting or seedling rootstock [6].

The use of soursop dates back to pre-Columbian times, where cultures such as the Maya used it for both consumption and tribute [7,8]. There is archaeological evidence of its consumption dating back to at least 3400 BC [9], with records in Peru associated with the Chimú culture around 1000 AD [10]. Over the centuries, soursop has been valued for its flavor and nutritional value, in addition to its relevance in traditional medicine. The fruits of *A. muricata* are consumed fresh or processed in products such as juices, ice creams, yogurts, and jams, being a rich source of vitamins, minerals, and dietary fiber [11]. Their pulp is rich in protein, carbohydrate, water, nonreducing sugar, and vitamins B1/B2 and C [12,13]. In traditional medicine, beverages made with different parts of the plant have been used to treat various conditions, including parasitic infections, fever, liver and digestive diseases, among others [14]. Additionally, antibacterial, anti-inflammatory, antioxidant, and antitumor properties are attributed to it, which are related to the presence of secondary metabolites such as tannins, saponins [15], polyphenols, alkaloids, fatty acids [16], and acetogenins [17,18]. These compounds play crucial roles in plant growth and survival under various environmental conditions [16].

The synthesis and accumulation of these metabolites are influenced by genetic and environmental factors, such as light, temperature, water availability, and soil characteristics, as well as stress factors, such as drought or flooding [19,20]. In addition, the variation in the production of these compounds may depend on the part of the plant, the stage of development, and its geographical origin [19,21].

In Mexico, the cultivation of *A. muricata* is favored by suitable geographic, climatic and environmental conditions. However, production is limited by several factors, such as phytosanitary problems, deficiencies in production technology, lack of identification of highly productive or desirable cultivars, and insufficient characterization of the gene pool still present in the natural environment, homegardens, or underutilized areas along local roads or grazing areas [18]. Most of the soursop plants in southern Mexico are found in homegardens. The boundaries of homegardens, situated in either rural or urban settings, are frequently delineated by physical barriers, which results in distinctive conditions with regard to soil, microclimate, and biotic life. Consequently, these enclosed ecosystems demonstrate marked differences when compared to the surrounding natural environment [22].

Homegardens play a fundamental role in functionality and sustainability, providing essential ecosystem services such as pollination, shelter for a wide range of fauna, and facilitating the exchange of genetic material between populations. In urban areas, homegardens are becoming increasingly important as they contribute to improving air quality, reducing carbon dioxide emissions and mitigating against the effects of urban heat islands, while providing fresh food and occasionally small income opportunities for homeowners [23,24]. Furthermore, homegardens are indispensable for the conservation and utilization of plant species diversity for various purposes.

Several studies have shown a high diversity of these home gardens even in small spaces [25,26], as well as species in different states of domestication (wild, semi-domesticated and domesticated), which contributes to resilience against pests, diseases and the effects of environmental changes. Thus, home gardens represent an opportunity to address constraints in the production and conservation of plants such as *A. muricata* in a more local and sustainable environment.

Although *A. muricata* is cultivated in various tropical regions, studies on its genetic diversity and the production of bioactive compounds in homegardens are limited, particularly in southern Mexico, where agroecological conditions may significantly influence these traits. Secondary metabolites of *A. muricata*, such as polyphenols, alkaloids, and flavonoids, have important applications in both traditional and modern medicine. However, there is a lack of knowledge regarding the influence of genetic and environmental factors on the synthesis and accumulation of these compounds in plants cultivated in homegardens. It is therefore imperative that these crops are genetically and phytochemically characterized in order to ensure their conservation and to optimize their use in medicinal and food products.

The present study aims to determine the genetic diversity and population structure of *A. muricata* in homegardens in southern Mexico, in addition to identifying and quantifying key bioactive compounds. This multidisciplinary approach will provide crucial information regarding the relationship between genetic diversity, the synthesis of bioactive compounds, and the specific environmental conditions present at the local level, therefore contributing to the knowledge that is required for the conservation and improvement of this species.

## Materials and methods

### Study area and plant material

**Fig 1:**
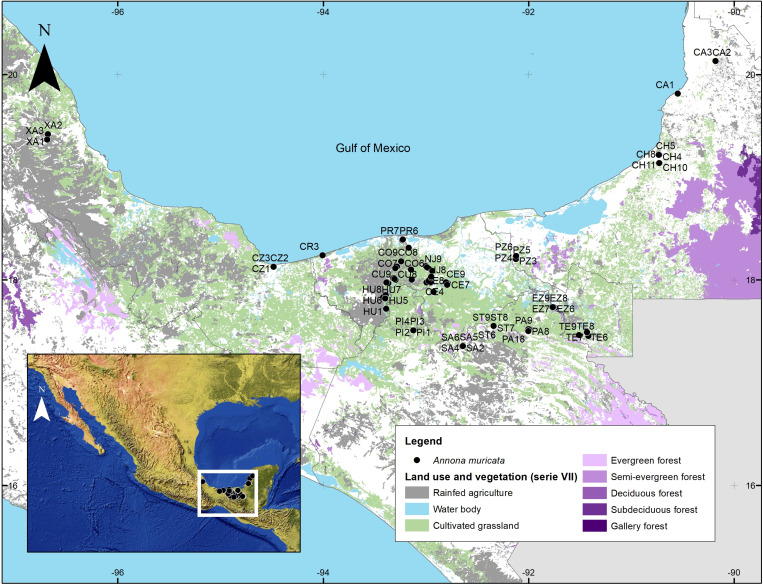
Geographical distribution of sampling sites of *A. muricata* L. in southern Mexico. XA: Xalapa, CZ: Coatzacoalcos, CR: Cardenas, HU: Huimanguillo, CU: Cunduacan, CO: Comalcalco, PR: Paraiso, PI; Pichucalco, NJ: Nacajuca, CE: Centro, SA: Sabanilla, ST: Salto de Agua, PZ: Palizada, PA: Palenque, EZ: Emiliano Zapata, TE: Tenosique, CH: Champotón, CA: Campeche. Source: INEGI (2021), Land Use and Vegetation Vector Dataset, Series VII, scale 1:250000. Downloaded from https://www.inegi.org.mx/temas/.

A total of 191 leaf tissue samples were collected from *A. muricata* L. trees in homegardens across various localities within the states of Veracruz, Tabasco, Chiapas, and Campeche (Fig 1 and Table 1), considering each locality as a distinct population. Inclusion criteria considered mature trees in optimal health, excluding those with visible signs of disease or environmental stress. To prevent duplications, each tree was assigned a unique code based on the locality and sample number, and all samples were adequately georeferenced (S1 Table).

**Table 1:** Information about sampling of *A. muricata* in southern Mexico and their use in our analyses.

State	Locality	Abbrev	N	Genetic	Chemical
Veracruz	Xalapa	XA	3	Yes	No
	Coatzacoalcos	CZ	3	Yes	No
Tabasco	Cardenas	CR	10	Yes	No
	Huimanguillo	HU	7	Yes	No
	Cunduacan	CU	20	Yes	Yes
	Comalcalco	CO	11	Yes	Yes
	Paraiso	PR	7	Yes	Yes
	Nacajuca	NJ	19	Yes	Yes
	Centro	CE	31	Yes	Yes
	Emiliano Zapata	EZ	11	Yes	Yes
	Tenosique	TE	13	Yes	No
Chiapas	Palenque	PA	17	Yes	Yes
	Pichucalco	PI	4	Yes	No
	Sabanilla	SA	5	Yes	No
	Salto de Agua	ST	13	Yes	Yes
Campeche	Champoton	CH	8	Yes	Yes
	Campeche	CA	3	Yes	No
	Palizada	PZ	6	Yes	Yes

Abbrev: abbreviation used in the manuscript, N: number of plants collected in each locality, Genetic: samples used in genetic analyses, Chemical: samples used in chemical analyses.

Ten leaves were collected per each tree, ensuring that there were no signs of physical damage, pests, or diseases. These were stored in silica gel bags for subsequent genetic analysis. For the purposes of chemical analyses, approximately 1 kg of fresh foliar tissue was collected from each locality/population. The collected plant tissue was dried in an oven (RSU Labsupply, Mexico City) at a temperature not exceeding 45°C and was subsequently ground using a Pulvex MPP300 mill.

### DNA extraction and microsatellite marker (SSR) analysis

DNA extractions were performed using the CTAB protocol with modifications following Viruel & Hormaza [27]. The quality and concentration of the extracted DNA were determined using a NanoDrop™ One spectrophotometer (Thermo Scientific, Waltham, MA, USA) by measuring absorbance at wavelengths of A260/280 nm and A260/230 nm. Ten microsatellites previously designed for *A. cherimola* and transferable to other species of the genus *Annona* [28,29] were used (Table 2).

**Table 2:** Characteristics of the SSRs loci used for *A. muricata.*

SSRs	Forward and reverse primers (5′–3′)	Repeat	Ta (°C)	Size (pb)	Expected size
LMCH5	F: CCCACTCTTCTACCCTCAAC	(CT)10	55	152-166	155-160
	R: CAAGTCCCTGTAAGAATCAGA				
LMCH6	F: GGCATCCTATATTCAGGTTT	(CT)14	55	204-224	220-254
	R: TTAAACATTTTGGACAGACC				
LMCH12	F: TATCTGCTTGAAACCAAAAC	(CT)17	55	152-167	152-168
	R: GCATTAGATGAGAAGGACTC				
LMCH21	F: TTTTATAGGAGGGGAGAGTAGA	(CT)18	55	159-172	159-172
	R: AAAACGACAACATTCCACAC				
LMCH29	F: GTACCATCTTTTAGGAAATC	(GA)9	55	189-193	185-195
	R: TGCAATCTATGTTAGTCAC				
LMCH39	F: AATTTGTATGGTGTTGACAG	(CT)11	55	119-123	187-195
	R: AGTTGTAGGTGGTTTAAGTTC				
LMCH42	F: TTTATCATTACGAGAGTTATCA	(GA)11	55	175-187	198-202
	R: AAAGTTGTCCTTTTACTCCT				
LMCH79	F: GAAGCAAGTAGACACGTAGTA	(CT)12	55	206-210	207
	R: AGGGTTGGTATTTCTTTATAGT				
LMCH87	F: AGTTAAGACACGAGATGATAAA	(GA)15	55	103-107	133-152
	R: CAAGTAAAGACTGAAAGGTTG				
LMCH118	F: AAAACTATAACCAGGAAGTAAA	(GA)10	55	221-239	221-239
	R: AGACTGATGGTCTTTTTCTC				

Ta: annealing temperature.

PCR reactions were carried out in a T100 Thermal Cycler (Bio-Rad Laboratories, Hercules, CA, USA) using the following temperature program: an initial step of 1 min at 94°C, 35 cycles of 30 sec at 94 °C, 30 sec at 55 °C, 1 min at 72 °C, and a final extension step of 5 min at 72 °C. The forward primers were labeled with fluorescence at the 5’ end, and the PCR products were detected and sized using a GeXp capillary DNA analysis system (Beckman Coulter Genome Lab). Samples were denatured at 90 °C for 120 s, injected at 2.0 kV for 30 s, and separated at 6.0 kV for 35 min. Samples with previously identified alleles were used as positive controls to ensure size accuracy and minimize variation among runs. Each PCR reaction and capillary electrophoresis was repeated at least twice to ensure the reproducibility of the results.

### Molecular data analysis

#### Genetic diversity.

The genetic diversity of the 18 populations of *A. muricata* was determined using the GENALEX 6.502 software [30] by calculating parameters such as the mean number of alleles (NA), the mean number of effective alleles (Ne), observed heterozygosity (*H*_*O*_), expected heterozygosity (*H*_*e*_), and fixation coefficient (*F*). Hardy-Weinberg equilibrium (HWE) was determined by calculating heterozygote deficiency using GENEPOP 4.0.10 [31]. CERVUS 3.0.7 [32] was used to calculate the polymorphic content (PIC). The presence of null alleles per locus was verified using the FREENA program [33].

#### Genetic structure.

The genetic structure was estimated using differentiation parameters, identification of substructures, and calculating genetic distance between the different populations. To determine the level of genetic differentiation, *F*_*ST*_ values were estimated using FREENA, which implements the ENA correction method to calculate *F*_*ST*_ values excluding null alleles [33]. Similarly, a molecular variance analysis (AMOVA) was calculated using GENALEX 6.502. To identify the population structure, STRUCTURE v2.3.1 software [34] was used, applying the Admixture model, which assumes that each sample has ancestry from one or more of genetically distinct sources (*K*) according to the run parameters [35]. The number of populations (*K*) was established from 1 to 20, with a burn-in period of 100,000 iterations followed by 1,000,000 Markov Chain Monte Carlo (MCMC) iterations and an individual alpha for each population set at 0.06 [35]. The determination of the most probable number of populations *K* was performed using the Evanno method [36] implemented on the STRUCTURE HARVESTER website [37]. Genetic distances were evaluated by estimating the Nei coefficient using GENALEX 6.502, and a dendrogram was constructed using the unweighted pair-group method with arithmetic mean (UPGMA) by the R program [38] and subsequently edited with the Interactive Tree Of Life (iTOL) program [39].

### Chemical data analysis

#### Preparation of extracts.

Samples from 10 locations (Table 1) were stored in resealable bags, in a suitable space protected from light and humidity. Drying was carried out in a Riossa Model H-102 culture oven, at a temperature of 35–40 °C with air recirculation for 24–48 h. The dried plant material was pulverized in a PULVEX-PLASTIC® mill (Series No. 101T0133M27) to obtain 4–6 mm particles. Maceration was carried out at room temperature, using ACS grade methanol (Meyer) to extract phenolic and flavonoids compounds, compatible with polar solvents. For the extraction of *A. muricata* leaves, a ratio of 1:3 (kg of dry plant material/L of solvent) was used, performing three macerations, with a 24 h rest followed by filtration. The solvent was recovered by distillation under reduced pressure in a rotary evaporator (Büchi® R-300, Switzerland). Finally, the extracts were freeze-dried (LABCONCO® freeze dryer 18) and stored at -4 °C.

#### Total flavonoid content (TFC).

The determination of flavonoids was carried out using a colorimetric method described by Liu [40]. Absorbance measurements were taken at 510 nm using a GENESYS™ 10 UV-Vis spectrophotometer (Thermo Scientific, Waltham, MA, USA), comparing the results with a blank prepared at the same wavelength. The standard used was rutin, and each assay was performed in triplicate. Flavonoid content was quantified in mg rutin equivalents (RE)/g of methanolic extract for the 10 populations.

#### Total phenolics content (TPC).

The determination of total polyphenols was carried out using the Folin-Ciocalteu method [41]. Absorbance measurements were performed at 760 nm, using gallic acid as reference reagent. Polyphenol content was expressed as mg gallic acid equivalents (GAE)/g of methanolic extract, also for the 10 populations.

#### Calibration curves.

Pre-calibration curves were prepared for both methods, using routine concentrations (0.01–0.05 mg/mL) for flavonoids and gallic acid (0.001–0.007 mg/mL) for polyphenols. Sample readings were measured at the same wavelengths as the standards, and the results were expressed as rutin equivalents (RE)/g or gallic acid (GAE)/g of methanolic extract.

Statistical analysis was performed using a one-way ANOVA to determine significant differences in the amount of secondary metabolites between the populations analyzed. A post-hoc multiple range test was performed using the Tukey/Kramer test. All statistics were performed using Statgraphics Centurion® V18 software with a confidence limit of α = 0.05. Before analysis, normality and homoscedasticity assumptions were checked [42]. Each assay was performed in triplicate.

### Relationship between genetic characterization and chemical compounds

To evaluate the possible relationship between the genetic profile of the population and their chemical characterization, we processed a multidimensional scaling (MDS) analysis using STATISTICA software. This analysis permits the reduction of a multidimensional dataset into a smaller dimensional space with a graphical visualization of data relationships for better understanding. For this analysis, we considered only the 10 populations for which chemical results (total polyphenol and flavonoid) were available. Data were categorized as follows: chemical content per population was classified into four categories based on percentiles: < 25 percentile, between 25 percentile and mean, between mean and 75 percentiles, and > 75 percentile. For the genetic group, we considered the dominant genetic profile for each population. Additionally, we considered the level of human disturbance on each site characterized by three levels ranging from highly perturbed (HD+++) to low perturbed (HD+) and the soil characteristics were categorized based on the National INEGI (National System of Statistical and Geographical Information; http://en.www.inegi.org.mx/temas/edafologia/) edaphology classification considering the World Reference Base for Soil Resource (Table 1.2 in IUSS Working Group WRB, 2022).

## Results

### Genetic analysis

#### Genetic diversity.

The 10 loci used for SSR analysis were effective in detecting the genetic diversity of *A. muricata*, including loci previously reported as monomorphic in *A. cherimoya*, such as the LMCH21 and LMCH118 markers. A total of 61 alleles were observed, with the detection of 19 unique alleles. The Centro locality showed the highest number of unique alleles with 5 alleles, followed by Cunduacan and Champoton with 3 unique alleles each. Overall, the Centro population displayed the highest values of genetic diversity, while the Pichucalco population exhibited the lowest (Table 3). The Xalapa population demonstrated the highest value of *H*_*o*_ whereas Huimanguillo displayed the lowest. The expected heterozygosity (*H*_*e*_) among the 18 populations analyzed ranged from 0.28 to 0.52, with a mean of 0.41. The global Polymorphic Information Content (PIC) value was 0.53, which corresponds to a moderately polymorphic value. The Centro population demonstrated the highest PIC value while the lowest value was observed in Pichucalco. The inbreeding coefficient varied considerably among populations ranging from highly negative values29/04/2025 (indicative of excess of heterozygotes) in some populations (XA, PR, PI) to highly positive values (indicative of inbreeding) for many populations (Table 3). No null alleles were found using the FreeNa software. The details of all genetic diversity parameters are presented in Table 3.

**Table 3:** Genetic diversity parameters for the 18 populations of *Annona muricata L.*

Population	*n*	*N*_*A*_	*N*_*e*_	*H*_*o*_	*H*_*e*_	*F*	*PIC*	*HWE*
XA	3	2.100	1.834	**0.467**	0.350	**-0.315**	0.298	NS
CZ	3	2.100	1.897	0.367	0.335	-0.037	0.284	NS
CR	10	3.200	2.391	0.408	0.522	0.257	0.468	***
HU	8	2.600	1.897	0.271	0.407	**0.456**	0.353	***
CU	20	3.000	1.917	0.298	0.417	0.424	0.355	***
CO	11	2.600	2.099	0.297	0.409	0.308	0.357	***
PR	7	2.300	2.041	0.429	0.384	-0.106	0.330	***
NJ	19	2.500	1.845	0.269	0.349	0.332	0.301	***
CE	30	**3.900**	**2.520**	0.386	**0.528**	0.271	0.479	***
EZ	11	2.700	2.113	0.427	0.476	0.102	0.405	***
TE	13	2.800	1.969	0.433	0.441	0.127	0.371	***
PA	17	3.200	2.447	0.395	0.519	0.257	0.463	***
PI	4	**1.700**	**1.583**	0.350	**0.281**	-0.257	0.223	NS
SA	5	1.900	1.756	**0.260**	0.349	0.328	0.283	*
ST	13	3.000	2.285	0.337	0.459	0.320	0.412	***
CH	8	2.200	1.614	0.350	0.323	-0.015	0.269	***
CA	3	1.800	1.620	0.267	0.328	0.238	0.258	NS
PZ	6	2.000	1.725	0.408	0.345	-0.067	0.286	NS
Global	191	2.533	1.975	0.357	0.401	0.166	0.533	***

n = number of individuals, *N*_*A*_ = mean number of alleles, *N*_*e*_ = mean number of effective alleles, *H*_*O*_ = observed heterozygosity, *H*_*e*_ = expected heterozygosity, *F* = inbreeding coefficient, *PIC* = Polymorphic information content, *HWE* = Hardy–Weinberg equilibrium, NS = not significant, * *P*<0.05, *** *P*<0.001. Bold values indicate lower and higher values. For abbreviations of populations see Table 1.

#### Genetic structure.

Populations showed moderate genetic differentiation with *F*_*ST*_ values ranging between 0.05 to 0.25, regardless of ENA adjustment [33]. Two pairwise populations (CA-CE and CA-ST) exhibited pronounced genetic differentiation, with *F*_*ST*_ values of 0.29 and 0.26 respectively (S2 Table). The Analysis of Molecular Variance (AMOVA) revealed that 64% of the variance is within individuals, 30% among individuals, and 6% among populations. These findings indicate low genetic differentiation between populations (S3 Table).

**Fig 2:**
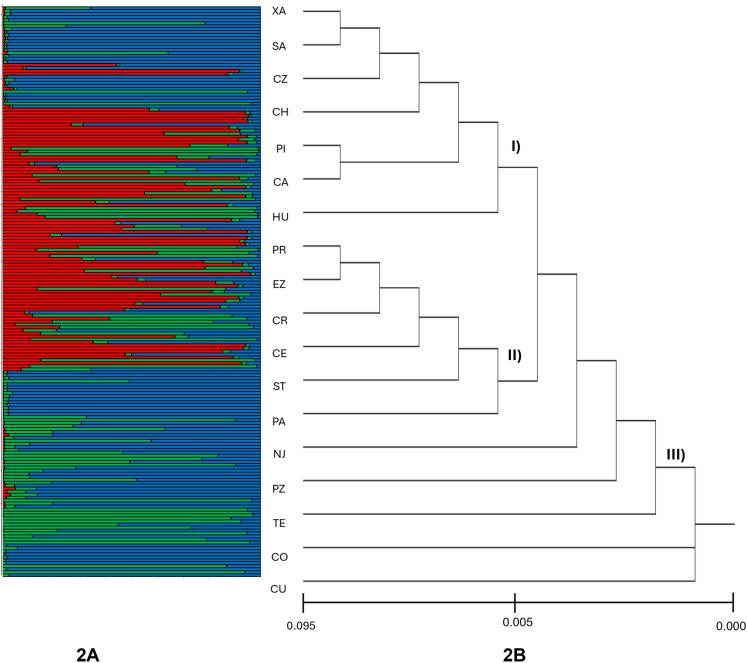
Genetic structure of 18 populations of *A. muricata* in Southeastern Mexico. (A) clusters (K = 3) estimated from a Bayesian genetic partitioning evaluation using STRUCTURE. Each individual is represented by a horizontal-colored line. **(B)** UPGMA based on Nei genetic distance. XA: Xalapa, CZ: Coatzacoalcos, CR: Cárdenas, HU: Huimanguillo, CU: Cunduacán, CO: Comalcalco, PR: Paraíso, PI; Pichucalco, NJ: Nacajuca, CE: Centro, SA: Sabanilla, ST: Salto de Agua, PZ: Palizada, PA: Palenque, EZ: Emiliano Zapata, TE: Tenosique, CH: Champotón, CA: Campeche. Roman numerals indicate the identified groupings.

Population structure was assessed using the Evanno method, based on probability assignments estimated by STRUCTURE. The analysis suggested that the most probable number of populations is *K* = 3 represented by three different colors, blue, red, and green (S1 Fig and S4 Table). Initially, the populations were sorted by their geographic proximity from west to east, to facilitate the identification of potential patterns. However, no clear geographic relationship could be determined among the three identified genetic groups. Therefore, they were subsequently ordered based on the results of the genetic distance tree (Fig 2). Overall, there was a pronounced predominance of affiliation with the blue cluster in locations such as Veracruz (XA, CZ), Tabasco (CO), and Chiapas (SA, ST). Conversely, other locations exhibited a certain degree of mixture among the three clusters (Fig 2A).

The genetic distance tree, constructed using the UPGMA method, revealed the presence of three distinct groups (Fig 2B). The nodes in the dendrogram represent the fusion of two population groups, while the distance between these nodes reflects the genetic similarity between the groups. The first group (I) includes the populations XA, SA, CZ, CH, PI, CA and HU; the second group (II) includes PR, EZ, CR, CE, ST and PA; In contrast, the NJ, PZ, TE, CO and CU populations do not exhibit a specific genetic association. It is noteworthy that the distance between the nodes of groups I and II is relatively small, suggesting a close genetic relationship between them. In contrast, the distance between the nodes of group II and the populations that could not be grouped is greater, indicating a lower genetic connection between these sets.

### Total flavonoid and polyphenol content

**Fig 3:**
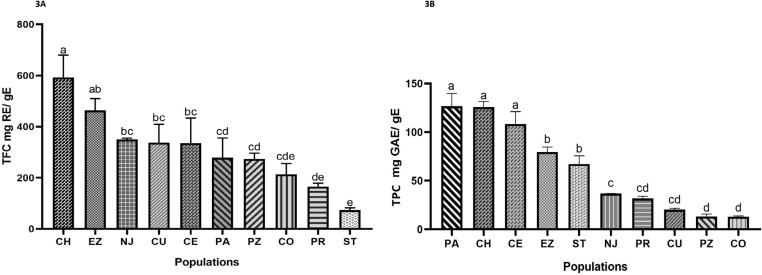
Flavonoid and polyphenol content in populations of *A. muricata.* **(A)** Flavonoid content expressed in mg rutin equivalent/g of extract. **(B)** Polyphenol content expressed in mg gallic acid equivalent/g of extract. Values are presented as means ± standard deviation (SD) based on three repetitions. Results of post-hoc Tukey test, where different letters (a, b, c, d) indicate statistically significant differences between groups (*P* < 0.05); populations with the same letter are not significantly different. PA: Palenque, CH: Champotón, CE: Centro, EZ: Emiliano Zapata, ST: Salto de Agua, NA: Nacajuca, PR: Paraíso, CU: Cunduacán, PZ: Palizada, CO: Comalcalco.

Results of total flavonoid and polyphenol contents are shown in Fig 3, significant difference in flavonoid (*F* = 19.69, DF = 29, *P* < 0.0001) and polyphenol (*F* = 130.13, DF = 29, *P* < 0.0001) content was observed among all populations, with values ranging from 73.48 to 592.70 mg RE/gE for flavonoids and from 13.10 to 126.59 mg GAE/gE for polyphenols. Regarding flavonoid contents, distinct categories could be observed (Fig 3A). The highest concentrations of flavonoids were observed in CH and EZ, with values of 592.70 and 464.14 mg RE/gE, respectively. A group comprising NJ, CU, CE, PA, and PZ, showed moderate concentrations of flavonoids with values ranging from 273.57 to 349.80 mg RE/gE. The lowest concentrations of flavonoids were observed in the populations CO, PR, and ST, with values ranging between 75.48 and 213.50 mg RE/gE. The highest concentrations of polyphenols (Fig. 3B) were observed in PA, CH, and CE, with values varying between 108.32 and 126.58 mg GAE/gE. Two populations, ST and EZ, exhibited lower concentrations, with values ranging from 67.20 to 79.74 mg GAE/gE, followed by the populations NJ, PR, and C, with values varying between 20.14 and 36.67 mg GAE/gE). The lowest levels of polyphenols were observed in the populations PZ and CO with values varying between 13.10 and 13.12 mg GAE/gE.

### Relationship between genetic characterization and chemical compounds

**Fig 4:**
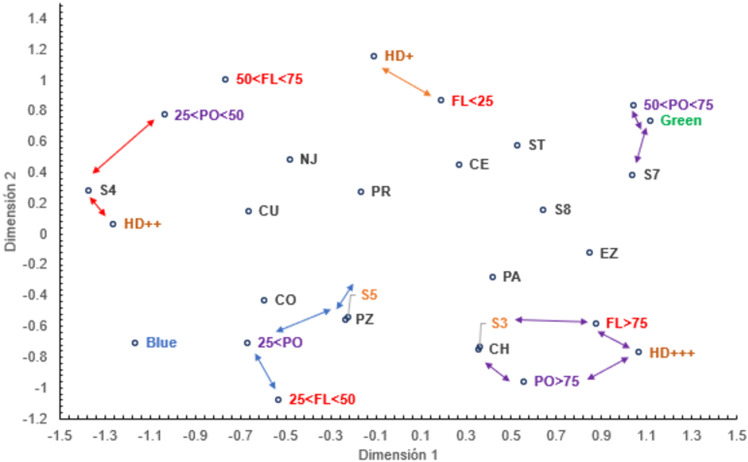
Multidimensional scaling analysis (MDS) showing the distribution of a complex dataset for *A. muricata* in south Mexico. Blue and green: dominant genetic profile of each population, FL: flavonoid content, PO: polyphenol content, HD: human disturbance (from high: +++ to low: +), S3-S4-S5-S7-S8: soil classification, and codes for population: PA (Palenque), CH (Champoton), CE (Centro), EZ (Emiliano Zapata), ST (Salto de Agua), NA (Nacajuca), PR (Paraíso), CU (Cunduacan), PZ (Palizada), CO (Comalcalco).

MDS analysis highlights some positive relationships (Fig 4). Blue and green genetic profiles from the structure results are separated along the X-axis suggesting some correlation with the chemical profile. Specifically, the genetic blue profile appears to be associated with a low chemical content (polyphenol < 25 and 25 <flavonoid < 50) while the green genetic profile is typified by a medium-high polyphenol content. Furthermore, there appears to be a relationship between human disturbance and chemical content. For example, highly disturbed sites appear to be associated with very high chemical content (flavonoids and polyphenols > 75). The genetic and phytochemical data are available as supplementary material (S5 Table).

## Discussion

Our results highlighted an interesting relationship among phenolic composition, genetic composition, and environmental/human factors for *A. muricata*, an important economic resource worldwide. In the study area, three genetic groups of *A. muricata* were identified with moderate genetic diversity (*H*_e_ ranges from 0.28 to 0.52). Regarding phytochemical compounds, significant differences were found in flavonoid and total polyphenol levels among the populations, with Champoton and Emiliano Zapata standing out for their high flavonoid content, and Palenque, Champoton, and Centro for their polyphenol content. The observed relationship between genetic composition, secondary metabolite content, human activity, and environmental conditions suggests that genetic diversity may play a role in the accumulation of bioactive compounds, making it relevant for management and medicinal use. However, limitations of this study include the sample size or the lack of analysis of other environmental factors that may affect metabolite synthesis. Nevertheless, this study represents a starting point for further exploration of the complex relationships governing the production of secondary metabolites in *A. muricata*.

### Genetic diversity

The genetic diversity in the studied populations of *A. muricata* in family orchards of southeastern Mexico was moderate. The number of alleles (n = 61) found in 191 samples of *A. muricata* is low compared to values reported in other *Annona* species, such as those documented by [43] who found 156 alleles in 154 samples of *Annona senegalensis*, and by [44] with 176 alleles in 1765 accessions of *Annona cherimola* Mill. However, our values are higher than those reported by [45] with 30 alleles in 102 accessions of *A. muricata.* The overall *H*_e_ value of 0.40 indicates a low to moderate genetic diversity, which could be attributed to the fact that the *A. muricata* plants in our study were exclusively collected from homegardens. These homegardens are subject to selective pressures and specific cultural practices, and reflect the needs of the human groups that maintain them [26]. In addition, the fact that their cultivation is predominantly distributed through human intervention, results in the presence of plants from various geographical regions [46], further emphasizing the influence of culture and human action on the genetic composition of the plants studied. Furthermore, moderate heterozygosity also suggests a potential adaptive capacity of *A. muricata* populations. Despite the absence of substantial genetic variability, the diversity observed in our study enables plants to adapt effectively to specific environmental changes and local selective pressures [47–49]. This is particularly relevant in the context of climate change, where adaptive capacity could play a key role in the survival and development of this species. In addition, artificial selection and cultural management in homegardens have favored the maintenance of sufficient genetic diversity, reflecting a balance between adaptation to human needs and the ability of this plant species to maintain its adaptive potential [50]. In southern Mexico, these gardens may prioritize certain traits that are valuable for human production. However, this may lead to a reduction in genetic diversity over generations by limiting the natural genetic pool. Thus, homegarden genetic diversity reflects the changing needs and preferences of households rather than the broader genetic diversity found in wild populations. Although not particularly high, the genetic diversity observed in the homegardens is still valuable as it is well adapted to local conditions. A promising avenue for future research would be the introduction of new genetic pools with the objective of enriching homegarden diversity. In addition, comparing genetic diversity within these homegardens with that of wild populations would be valuable, although, to our knowledge, such populations are not present in our study area.

The observed genetic diversity values were consistent with those reported for other Latin American populations of Annonaceae species. For example, genetic diversity values based on SSR genetic markers were 0.49 and 0.57 respectively [28,44,51] for *A. cherimola.* A study based on the analysis of isoenzymes and conducted with samples from homegardens revealed a *H*_e_ of 0.47 for *A. squamosa* [52]. In contrast, studies on the genetic diversity of *A. muricata* in Nayarit, Mexico, indicate a markedly low level of genetic diversity, as evidenced by an observed heterozygosity (*He*) of 0.050 and 0.164. It seems plausible that these findings are attributable to the establishment of orchards using seeds from a small number of initial trees [53,54]. Reports on commercial cultivars of *A. muricata* in Colombia demonstrated a *H*_*e*_ of 0.34, indicating low genetic diversity [45].

The majority of studies on the Annonaceae family have focused on commercial crops, demonstrating significant variations in genetic diversity between plants originating from commercial and non-commercial environments. It is imperative that both the number of samples evaluated in each study and their origin are considered as two factors playing a fundamental role when analyzing and comparing genetic diversity. Non-commercial environments, such as homegardens, tend to exhibit broader genetic diversity compared to commercial plantations, which often exhibit low genetic diversity. This emphasizes the importance of non-commercial systems as repositories for the preservation of individuals or populations that possess characteristics fundamental to the conservation of the genetic material of species of the Annonaceae, as well as other crops [55,56].

In addition, we observed variations in genetic diversity across different localities. Populations that demonstrated higher genetic diversity of *A. muricata* such as Centro, Cardenas, and Palenque, are characterized by a high degree of anthropogenic disturbance and significant human movement, both of which could influence genetic variability. Centro and Cardenas are two populous cities in the state of Tabasco and Palenque is a major tourist city with a large population influx. All these urban areas are typified by fragmented landscapes composed of patches of natural vegetation. These elements could favor higher genetic diversity, either through potential gene flow driven by fruit exchange or due to favorable environmental conditions. Although one might expect urban homegardens to harbor low genetic diversity because of their limited space, this assumption is not always accurate. Mobility of people in these areas not only increases the number of species but also successfully contributes to the richness and genetic diversity of local species, enriching both local and regional biodiversity [57].

The low inbreeding coefficient observed (*F* = 0.16) suggests a low level of inbreeding within the studied populations. The fixation index *F* (also called inbreeding coefficient) revealed values of between -1 and +1. Values close to zero are expected for random mating, while significantly positive values indicate inbreeding [58]. In cases where there is an excess of observed heterozygotes, the inbreeding coefficient can be negative [59]. The presence of a low inbreeding coefficient suggests a lower probability of allele homozygosity and a greater genetic variability. This aspect is essential for the adaptability and fitness of the species, as it establishes a broader genetic foundation capable of adapting to environmental changes, combating diseases, and sustaining a genetically resilient population [60]. Furthermore, it is worth mentioning that homegardens play a significant role in maintaining high levels of plant genetic diversity, both inter and intra-specifically, particularly in terms of traditional crop varieties and local breeds [26].

The varieties present in these gardens have been selected and adapted to environmental conditions closely linked to the communities that develop and cultivate them [61]. The propagation of trees in most homegardens is typically achieved through the use of seeds, a method that often results in the formation of variable populations that are adapted to local environmental conditions and relatively low but stable yields. These and many other elements of homegardens, are closely associated with the traditional uses, habits, and celebrations of the people who developed them [25]. Each household adapts its garden according to specific economic strategies and needs, resulting in changes in species composition over time [57]. The number of native fruit trees in homegardens is limited due to their high spatial requirements, a factor that significantly influences genetic diversity in these environments [62].

Some urban populations such as Xalapa, Coatzacoalcos, Pichucalco, Sabanilla, Campeche, and Palizada show no significant values when applying HWE, probably due to the low number of individuals sampled in these localities. A low number of individuals in a population can led to unreliable estimates regarding HWE conclusions [60]. These populations also showed lower *H*_e_ values (from 0.28 to 0.35), which may also be related to sample size. In contrast, the remaining populations presented significant results regarding Hardy-Weinberg equilibrium suggesting the possibility that some of the HWE assumptions are being challenged in these populations. These results suggest that most of the evaluated populations are open populations with some degree of genetic flow between gardens within each locality.

### Genetic structure

The minimal genetic differentiation and genetic structure observed in *A. muricata* in this study likely results from a combination of factors, including human intervention, environmental conditions, and evolutionary processes. The adaptability of soursop to a wide range of environmental conditions suggests that it may have evolved into different genetic groups in response to variations in climate, soil characteristics, and resource availability. However, artificial selection emerges as a highly influential factor in shaping these populations. It is plausible that the relatively low number of alleles may be attributed to the exclusive collection of *A. muricata* plants from homegardens, where selective pressures may have influenced the genetic variability. Phenotypic selection driven by human preferences for desirable traits, such as fruit size, taste, pulp production, and pest resistance [63], is likely to have played a crucial role in the formation of different genetic groups in the species.

The existence of three genetic groups without a clearly defined structure, and evidence of intermixing in certain regions, suggests that human intervention may be a primary factor contributing to this absence of structure. The influence of phenotypic selection is probably more pronounced in homegardens, which tend to be small and relatively close to each other. This geographical proximity of homegardens implies that soursop populations in these areas are more susceptible to genetic mixing compared to those in more distant areas.

The ability of soursop seeds to be transported over long distances through human flow facilitates genetic mixing between populations in different geographical regions. The importance of semi-wild fruit trees, such as *A. muricata*, in the diet of local communities and, in some cases, the use of their fruits for local trade, facilitates the dispersal of their seeds [64].

### Total flavonoid and polyphenol content

Quantification of total polyphenols and flavonoids focused on evaluating the regional variability of these metabolites, analyzing the patterns associated with various biological effects. This approach allowed us to identify significant differences among the populations studied, suggesting that environmental and genetic factors may influence the concentration of these compounds, known for their antioxidant potential and their role in health [18].

The production of secondary metabolites in *A. muricata* plants across southern Mexico revealed significant variation in flavonoid and polyphenol content. Particularly, the CH population presents the highest levels of these secondary metabolites, followed by CE, EZ, and PA. Although, wide variations among populations may be the result of numerous factors, the influence of genetics is likely to be significant [65]. In this context, populations exhibiting higher metabolite content may harbor genes that stimulate higher production of flavonoids and polyphenols. Future studies should consider measuring metabolite production at the individual tree level to determine whether this variation is a characteristic of specific individuals or a broader population trait. This approach would help determine whether the observed differences are predominantly genetic, indicating individual variation, or whether they reflect environmental effects acting uniformly across the population within each region. Compounds found in *A. muricata* extracts include coumaric acid, rutin and kaempferol glycoside [64–67] among others. These compounds have been shown to possess diverse biological activities, including antimicrobial, antioxidant, anti-inflammatory, and anticarcinogenic properties [68–70]. These characteristics make them metabolites of interest for their therapeutic potential and their impact on human health, especially in the development of natural treatments for various diseases.

The prevalence of plants with genes promoting high metabolic content is more often observed in specific areas, shaped by the different biotic and abiotic conditions to which they are exposed, and influenced by human interaction [71]. Many of these places, characterized by significant fruit production, constitute crucial economic centers, large urban hubs, and well-preserved tourist destinations.

Another significant factor to consider when analyzing variations in secondary metabolite production is intrinsically related to the environmental context. The consideration of both biotic and abiotic factors, as well as the specific environmental stress conditions of each location, is essential [72]. This variability also aligns with environmental stress generated by anthropogenic interaction in regions experiencing more human flow. Regions exhibiting lower secondary metabolite concentrations correspondingly align with lower population density and less urbanization. This latter aspect could represent a significant determining factor in the production of secondary metabolites in plants in this region.

### Relationship between genetic characterization and chemical compounds

Multidimensional scaling analysis suggests a relationship among genetic profile, chemical composition, and human disturbance within *Annona muricata* populations in southern Mexico. MDS analysis reveals a clear separation between the blue and green genetic population structure profiles, indicating significant genetic differences between these populations. Furthermore, the blue genetic profile is more related to low chemical content, while the green genetic profile shows a higher association with medium to high content of polyphenols. These findings imply a genetic basis for variations in chemical composition, suggesting that specific genes can promote the production of compounds with different chemical content levels [20]. Interestingly, highly disturbed sites seem to be linked to a higher chemical content, indicating that human disturbance can influence the chemical profile of a population [73]. It is plausible that human disturbance may favor the production of compounds with high content, potentially leading to the accumulation of compounds with antioxidant properties in these high-production areas. Although factors such as light and temperature were not directly assessed, it is probable that they influence the expression of genes related to secondary metabolite biosynthesis, as has been observed in species such as *Arabidopsis thaliana* [74], *Pyrus pyrifolia* [75], *Litchi chinensis* Sonn [76], and *Actinidia chinensis* [77]. In these cases, it has been shown that light and temperature can activate or inhibit the expression of transcription complexes, which in turn impacts the accumulation of secondary metabolites [78], suggesting an environmental interaction that could also be present in *A. muricata*. Another hypothesis is that artificial selection in homegardens may eliminate plants with specific characteristics, such as low chemical content, as they might be more susceptible to diseases or predation [56]. The interaction between genetic factors and human disturbance in the habitats where the study species thrives poses significant challenges for crop production. This scenario underlines the need to expand access to a broader range of plant genetic resources. In this context, species like *A. muricata*, cultivated in homegardens, play a crucial role in addressing the challenges faced by agricultural production [56].

## Conclusion

This study reveals that family gardens in Southeastern Mexico provide an important environment for the conservation of genetic diversity in *A. muricata* with a complex interrelationship between genetic composition, secondary metabolite diversity, and environmental factors. The influence of human activity, cultural practices, and environmental conditions shapes the genetic structure of the species, highlighting the importance of these non-commercial systems in preserving genetic variability.

Three genetic groups were identified, showing that most of the populations analyzed present moderate genetic diversity, with no clear evidence of geographic correlation. However, specific populations, such as Champotón, Palenque, Emiliano Zapata, and Centro, stood out for their high productivity of flavonoids and polyphenols, associated with moderate to high genetic diversity. This suggests that genetic richness in these populations could be driven by specific environmental conditions, such as land use and interaction with human communities.

While this study addresses chemical and genetic variation by considering variables such as soil type and land use, it is important to note that it does not explore the direct influence of specific environmental factors, such as the amount of light and temperature, which could also affect genetic diversity and the production of phytochemical compounds. Despite this limitation, the findings are significant, as they establish a basis for understanding the complex interactions between genetic and chemical characteristics of *A. muricata*. This research offers a valuable approach that can guide future studies to explore how specific environmental conditions influence the genetic richness and production of bioactive metabolites, which is crucial to maximize the therapeutic potential of this species in a context of climate change.

Furthermore, significant differences in the levels of bioactive compounds indicate that more disturbed environments may favor metabolite accumulation, highlighting an adaptive potential that is crucial for the survival of *A. muricata*. The identification of these populations, which show higher metabolite production and relevant genetic diversity, is fundamental to optimize new plantings and conserve the genetic diversity of this species in southern Mexico.

These findings emphasize the therapeutic potential of *A. muricata* and the need to implement management strategies that promote the sustainable use of this valuable plant. By integrating genetic tools into agricultural practices, the development of pharmaceutical products based on bioactive compounds can be fostered, benefiting both local communities and scientific research. This approach not only contributes to the understanding of the biodiversity of *A. muricata*, but also advocates for a sustainable model that respects and values natural resources in a context of global climate change. The approach used in this work can also be useful to optimize soursop management in other regions and could also be applied to other underutilized fruit crops.

## Supporting information

S1 Fig Determination of the optimal number of groups (clusters) using the method of Delta *K* implemented in Structure Harvester website. Results obtained from ten replicates for values of K from 1 to 20. (A) Ln P(D) method, and (B) ΔK method follow STRUCTURE HARVEST website.
(PDF)

S1 Table Location of the sampling sites of the A. muricata L. populations. Each sample with the identification number per individual used for the analyzes and geographical coordinates.
(PDF)

S2 Table FST genetic differentiation values among the 18 populations of Annona muricata analyzed. Values shown above with ENA correction, values below without ENA correction (Chapuis & Estoup, 2007).
(PDF)

S3 Table Analysis of molecular variance (AMOVA) for Annona muricata in southern Mexico. The AMOVA results indicated that the highest genetic differentiation occurred among individuals, accounting for 64% of the total genetic variation.
(PDF)

S4 Table Proportion of membership of each pre-defined population in each of the 3 clusters. The population structure, evaluated using the Evanno method and based on probability assignments estimated by the STRUCTURE program, suggests that the most probable number of populations is K = 3, represented by three distinct groups.
(PDF)

S5 Table Genetic and chemicals database used for this study. Concentration of chemical components are presented as means (of three repetitions) with its standard deviation.
(XLSX)
