# Correction: Genetic analysis and phytochemical profile of soursop (*Annona muricata* L.) cultivated in family orchards in southeastern Mexico

**DOI:** 10.1371/journal.pone.0332514

**Published:** 2025-09-15

**Authors:** Heidi Beatriz Montejo-Mendez, Julia Maria Lesher-Gordillo, Jose I. Hormaza, Carlos Ernesto Lobato-Garcia, Abraham Gomez-Rivera, Salima Machkour-M’Rabet, Manuel Ignacio Gallardo-Alvarez, Nerea Larranaga, Aminta Hernandez-Marin, Alejandra Valdes-Marin, Ricardo Lopez-Rodriguez, Yann Henaut, Hilda María Díaz-Lopez

The following information is missing from the Funding statement: Support was also obtained from LINCGLOBAL program of CSIC (LINCG24052).

## References

[1] Montejo-Mendez HB, Lesher-Gordillo JM, Hormaza JI, Lobato-Garcia CE, Gomez-Rivera A, Machkour-M’Rabet S, et al. Genetic analysis and phytochemical profile of soursop (*Annona muricata* L.) cultivated in family orchards in southeastern Mexico. PLoS One. 2025;20(5):e0321846. doi: 10.1371/journal.pone.0321846 40333692 PMC12057873

