# Supplementary figures and images for: Genetic analysis and phytochemical profile of soursop (Annona muricata L.) cultivated in family orchards in southeastern Mexico
Source: PLoS One. 2025 May 7;20(5):e0321846. doi: 10.1371/journal.pone.0321846 (PMC12057873; doi:10.1371/journal.pone.0321846)

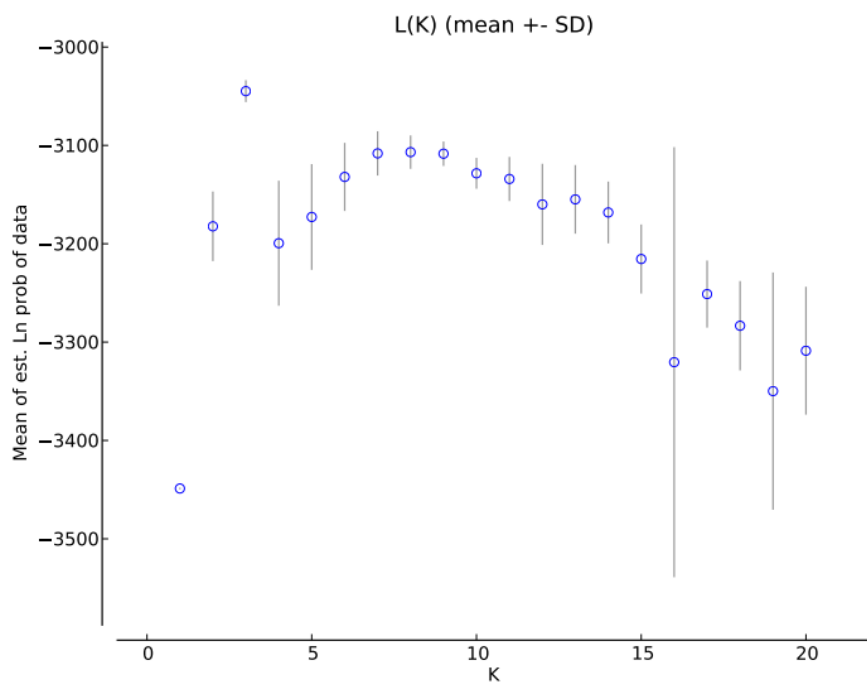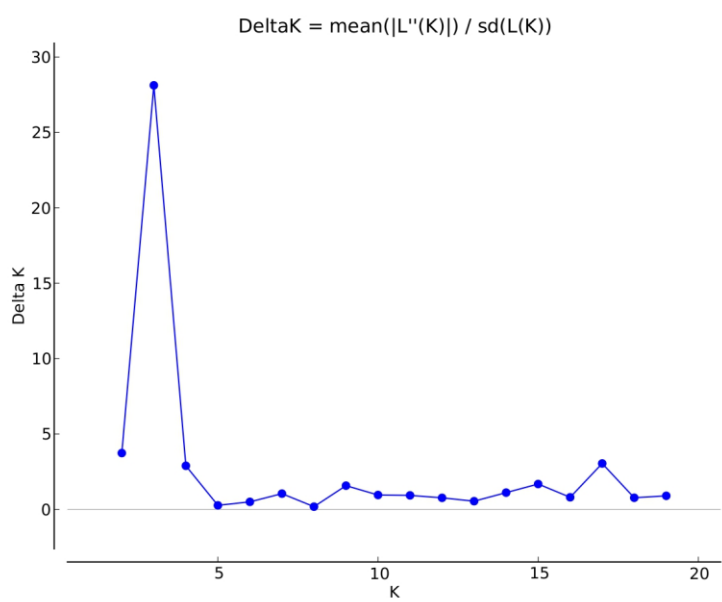

Supplement: S1 Fig — Results obtained from ten replicates for values of K from 1 to 20. (A) Ln P(D) method, and (B) ΔK method follow STRUCTURE HARVEST website. (PDF) [file pone.0321846.s001.pdf]
