# Supplementary material for: Genetic analysis and phytochemical profile of soursop (Annona muricata L.) cultivated in family orchards in southeastern Mexico
Source: PLoS One. 2025 May 7;20(5):e0321846. doi: 10.1371/journal.pone.0321846 (PMC12057873; doi:10.1371/journal.pone.0321846)
Supplement: S1 Table — Each sample with the identification number per individual used for the analyzes and geographical coordinates. (PDF) [file pone.0321846.s002.pdf]

State	SSR analysis	Chemical analysis	Code	Latitude	Longitude
Veracruz	Xalapa (XA)	-	XA1	19,419,306	96,680,639
			XA2	19,420,778	9,668,075
			XA3	19,367,722	96,687,361
	Coatzacoalcos (CZ)	-	CZ1	18,129,333	94,481,833
			CZA2	18,128,806	94,483,861
			CZA3	18,128,556	94,483,583
Tabasco	Cardenas (CR)	-	CR1	1,800,025	93,295,278
			CR2	18,000,778	93,295,389
			CR3	18,241,194	94,006,222
			CR4	179,765	93,384,833
			CR5	17,966,917	93,363,472
			CR6	17,969,361	9,336,575
			CR7	17,981,806	93,381,472
			CR8	17,976,972	93,384,639
			CR12	18,018,667	93,317,806
			CR13	18,019,167	93,308,917
	Huimanguillo (HU)	-	HU7	17,720,972	93,386,472
			HU8	178,325	934,155
			HU1	17,851,944	93,390,611
			HU2	17,822,861	93,395,278

			HU3	178,215	93,393,972
			HU4	17,822,583	93,392,778
			HU5	17,820,583	93,397,361
			HU6	17,821,806	93,396,528
	Cunduacan (CU)	Cunduacan	CU1	18,103,417	93,147,667
			CU2	18,104,944	93,148,889
			CU3	18,102,694	93,144,889
			CU4	18,102,694	93,144,944
			CU5	18,102,889	93,144,778
			CU6	18,102,389	93,144,556
			CU7	18,010,056	93,305,417
			CU8	18,010,083	93,305,306
			CU9	18,010,194	93,305,028
			CU10	18,004,028	93,134,306
			CU11	18,004,028	93,134,306
			CU12	18,004,194	93,134,306
			CU13	18,004,556	931,365
			CU14	18,004,556	93,136,722
			CU15	18,004,333	93,136,778
			CU16	18,133,083	93,282,306
			CU17	181,325	93,282,556
			CU18	18,132,611	93,282,583
			CU19	18,115,611	9,329,675

			CU20	18,115,778	93,296,667
	Comalcalco (CO)	Comalcalco	CO1	1,818,225	93,240,639
			CO2	18,182,194	93,240,694
			CO3	18,182,278	93,240,694
			CO4	18,181,917	93,240,694
			CO5	18,181,833	9,324,075
			CO6	18,181,972	93,240,556
			CO7	18,181,889	93,240,611
			CO8	18,182,167	93,240,972
			CO9	18,182,889	93,239,806
			CO10	18,182,889	93,239,861
			CO11	18,182,972	93,239,778
	Paraíso (PR)	Paraíso	PR1	18,314,444	93,167,667
			PR2	18,314,556	93,167,778
			PR3	18,314,667	93,167,667
			PR4	183,145	93,167,806
			PR5	1,838,775	93,230,694
			PR6	18,393,167	93,226,972
			PR7	18,393,667	93,225,333
	Nacajuca (NJ)	Nacajuca	NJ1	18,089,694	92,939,167
			NJ2	18,089,528	92,938,167
			NJ5	18,139	93,003,028
			NJ6	18,139	93,002,917

			NJ7	18,136,861	93,001,583
			NJ8	18,136,889	93,001,611
			NJ9	18,119,028	92,983,778
			NJ10	18,119	92,983,556
			NJ11	18,125,139	9,299,025
			NJ12	18,125,056	92,989,361
			NJ13	18,124,917	92,989,472
			NJ14	18,124,861	92,989,333
			NJ15	1,812,475	92,989,333
			NJ16	18,124,806	92,989,667
			NJ17	18,124,944	92,990,056
			NJ20	18,021,806	92,947,722
			NJ21	18,033,056	92,948,083
			NJ22	18,033,083	92,947,556
			NJ23	18,033	92,947,333
	Centro (CE)	Centro	CE1	17,977,389	92,957,611
			CE2	17,954,528	92,791,222
			CE3	17,959,889	92,785,944
			CE4	17,969,333	92,790,278
			CE5	17,975,306	92,786,528
			CE6	17,976,472	92,786,972
			CE7	17,976,167	92,787,472
			CE8	17,976,389	92,786,694

			CE9	17,971,222	92,788,111
			CE10	17,971,268	92,787,982
			CE11	17,973,417	92,789,028
			CE12	17,973,463	92,788,609
			CE13	17,972	92,786,778
			CE14	1,797,331	92,789,264
			CE15	17,979,861	92,800,556
			CE16	17,979,861	92,804,278
			CE17	179,795	92,803,667
			CE18	17,979,389	92,804,028
			CE19	17,883,639	92,924,333
			CE20	1,788,375	92,924,306
			CE21	17,883,889	92,925,028
			CE22	17,883,889	92,924,944
			CE23	17,975,806	92,994,028
			CE24	17,975,778	92,994,667
			CE25	17,990,333	92,973,778
			CE26	17,990,472	92,973,806
			CE27	17,953,778	92,792,639
			CE29	17,953,472	92,792,528
			CE30	17,953,694	92,792,222
			CE31	17,953,889	92,791,944
	Emiliano Zapata (EZ)	Emiliano Zapata	EZ1	17,736,917	91,754,333

			EZ2	17,731,667	91,744,917
			EZ3	17,731,667	91,744,917
			EZ4	17,731,667	91,744,917
			EZ5	17,737,139	91,755,222
			EZ6	17,737,083	91,755,333
			EZ7	17,738,028	91,76
			EZ8	17,738,167	91,759,861
			EZ9	17,738,167	91,759,861
			EZ10	177,385	91,761,361
			EZ11	17,737,139	91,765,917
	Tenosique (TE)	-	TE1	17,456,583	91,417,528
			TE2	17,456,167	91,417,278
			TE3	17,491,222	91,431,222
			TE4	1,749,075	91,430,778
			TE5	17,491,722	91,432,583
			TE6	17,490,417	91,430,722
			TE7	17,493,167	91,431,056
			TE8	17,462,333	91,501,944
			TE9	17,461,722	91,499,944
			TE10	17,463,389	91,502,639
			TE11	17,465,972	91,509,528
			TE12	1,746,575	91,510,694
			TE13	17,465,417	91,508,361

Chiapas	Palenque (PA)	Palenque	PA1	17,512,111	92,006,611
			PA2	17,512	92,006,528
			PA4	17,514,417	91,999,917
			PA5	17,514,417	91,999,917
			PA6	17,518,556	91,994,111
			PA7	17,519,528	9,200,075
			PA8	17,520,028	92,000,778
			PA9	17,519,972	92,001,333
			PA10	17,518,806	92,001,694
			PA11	17,516,667	91,997,167
			PA12	17,521,833	91,997,778
			PA13	17,524,083	91,995,639
			PA14	17,524,083	91,995,639
			PA15	17,522,028	92,002,417
			PA16	17,504,722	92,002,722
			PA17	17,504,722	92,002,361
			PA18	17,504,583	92,002,389
	Pichucalco (PI)	-	PI1	17,507,306	93,123,722
			PI2	17,507,306	93,123,722
			PI3	17,511,306	93,122,444
			PI4	17,511,306	93,122,444
	Sabanilla (SA)	-	SA1	17,362,306	92,640,111
			SA2	17,358,056	92,64

			SA3	17,358,111	92,638,528
			SA4	17,357,306	92,639,472
			SA5	17,357,167	92,639,472
	Salto de Agua (ST)	Salto de Agua	ST1	17,551,778	92,337,056
			ST2	17,551,778	92,337,083
			ST3	17,551,778	92,337,028
			ST4	17,551,833	92,337,111
			ST5	17,552,083	92,337,361
			ST6	17,552,111	92,337,417
			ST7	17,550,944	92,339,417
			ST8	17,550,861	92,339,333
			ST9	17,550,833	92,340,028
			ST10	17,551,083	92,340,194
			ST11	17,551,139	92,340,361
			ST12	17,551,167	923,405
			ST13	17,554,361	92,339,917
Campeche	Champotón (CH)	Champotón	CH1	19,222,972	90,731,056
			CH2	19,221,111	90,729,944
			CH3	19,224	9,073,025
			CH4	19,219,139	90,727,917
			CH5	19,219,972	90,731,583
			CH8	19,138,833	9,072,775
			CH10	19,139,028	90,726,722

			CH11	19,139,028	90,726,972
	Campeche (CA)	-	CA1	19,815,056	90,546,722
			CA2	20,130,083	90,178,667
			CA3	20,132,139	90,178,389
	Palizada (PZ)		PZ1	18,12,02.1	92,07,24.8
			PZ2	18,12,05.1	92,07,24.2
			PZ3	18,12,06.5	92,07,24.7"
			PZ4	18,12,17.5	92,07,24.2
			PZ5	18,12,17.7	92,07,24.3
			PZ6	18,14,04.8	92,07,15.0
