# Supplementary material for: Genetic analysis and phytochemical profile of soursop (Annona muricata L.) cultivated in family orchards in southeastern Mexico
Source: PLoS One. 2025 May 7;20(5):e0321846. doi: 10.1371/journal.pone.0321846 (PMC12057873; doi:10.1371/journal.pone.0321846)
Supplement: S2 Table — Values shown above with ENA correction, values below without ENA correction (Chapuis & Estoup, 2007). (PDF) [file pone.0321846.s003.pdf]

	CA	PZ	CH	PA	PI	SA	ST	CE	NJ	CR	PR	CU	HU	CO	EZ	TE	CX	XA
CA		0.0000	0.1315	0.0870	0.1553	0.2130	0.2620	0.2948	0.1528	0.1726	0.0337	0.1615	0.1880	0.1421	0.2226	0.0946	0.0914	0.2183
PZ	0.0000		0.0695	0.0000	0.0724	0.1290	0.1771	0.1279	0.0158	0.0997	0.0000	0.0698	0.0789	0.0872	0.2144	0.0584	0.0000	0.0355
CH	0.0712	0.0000		0.0042	0.0607	0.0905	0.0569	0.0802	0.1062	0.0000	0.0815	0.0000	0.1208	0.0000	0.0238	0.0541	0.1301	0.0737
PA	0.0360	0.0000	0.0000		0.0021	0.0864	0.0789	0.0738	0.0099	0.0120	0.0000	0.0104	0.0585	0.0125	0.1062	0.0119	0.0298	0.0396
PI	0.1289	0.0325	0.0560	0.0000		0.0653	0.1331	0.1408	0.0314	0.0704	0.0534	0.0841	0.0383	0.0625	0.1173	0.0246	0.0838	0.0503
SA	0.1760	0.0797	0.0556	0.0510	0.0446		0.2195	0.2393	0.1197	0.1280	0.1284	0.1437	0.1135	0.1133	0.1727	0.0796	0.1851	0.1405
ST	0.2336	0.1366	0.0332	0.0665	0.1293	0.1902		0.0894	0.1887	0.0586	0.1949	0.0396	0.2470	0.1014	0.0889	0.1532	0.1493	0.1425
CE	0.2779	0.0806	0.0224	0.0220	0.1134	0.1864	0.0790		0.1454	0.0984	0.2044	0.0648	0.2486	0.1063	0.1551	0.1650	0.1105	0.0949
NJ	0.1345	0.0000	0.0848	0.0000	0.0268	0.0980	0.1842	0.1111		0.1011	0.0057	0.0878	0.0406	0.0974	0.2108	0.0483	0.0522	0.0894
CR	0.1185	0.0415	0.0000	0.0212	0.0724	0.1045	0.0354	0.0509	0.0866		0.0856	0.0000	0.1188	0.0026	0.0787	0.0762	0.1338	0.0990
PR	0.0000	0.0000	0.0399	0.0000	0.0371	0.0970	0.1673	0.1527	0.0000	0.0581		0.0697	0.0477	0.0717	0.1990	0.0354	0.0477	0.1689
CU	0.1008	0.0001	0.0000	0.0091	0.0858	0.1098	0.0241	0.0223	0.0688	0.0000	0.0301		0.1082	0.0025	0.1012	0.0797	0.1080	0.0734
HU	0.1827	0.0582	0.0706	0.0038	0.0046	0.0548	0.2292	0.2269	0.0172	0.0702	0.0089	0.0652		0.0894	0.2487	0.0555	0.1387	0.0829

**CO**	0.0823	0.0285	0.0000	0.0118	0.0562	0.0801	0.0855	0.0622	0.0772	0.0000	0.0338	0.0000	0.0342		0.0753	0.0528	0.1364	0.0931
**EZ**	0.1711	0.1560	0.0143	0.0934	0.1078	0.1425	0.0461	0.1044	0.1951	0.0647	0.1644	0.0823	0.2075	0.0657		0.1173	0.1997	0.1954
**TE**	0.0771	0.0343	0.0493	0.0017	0.0072	0.0519	0.1672	0.1630	0.0359	0.0787	0.0214	0.0825	0.0238	0.0466	0.1177		0.0971	0.1087
**CX**	0.0748	0.0000	0.0859	0.0000	0.0609	0.1574	0.1344	0.0791	0.0337	0.1004	0.0167	0.0725	0.1252	0.0987	0.1607	0.0906		0.1088
**XA**	0.1667	0.0000	0.0301	0.0000	0.0114	0.0861	0.1152	0.0546	0.0523	0.0605	0.0842	0.0337	0.0247	0.0509	0.1477	0.0804	0.0382	

0-0.05 Indicate low genetic differentiation.

0.05-0.25 Indicate moderate genetic differentiation.

> 0.25 Represent pronounced levels of genetic differentiation.
