# Supplementary material for: Genetic analysis and phytochemical profile of soursop (Annona muricata L.) cultivated in family orchards in southeastern Mexico
Source: PLoS One. 2025 May 7;20(5):e0321846. doi: 10.1371/journal.pone.0321846 (PMC12057873; doi:10.1371/journal.pone.0321846)
Supplement: S3 Table — The AMOVA results indicated that the highest genetic differentiation occurred among individuals, accounting for 64% of the total genetic variation. (PDF) [file pone.0321846.s004.pdf]

Source	df	SS	MS	Est. Var.	%	***F*-Statistics**	Value	***P***
**Among populations**	17	114.851	6.756	0.168	6%	F_{st}	0.064	0.001
**Among individuals**	173	566.453	3.274	0.799	30%	F_{is}	0.323	0.001
**Within individuals**	191	320.000	1.675	1.675	64%	F_{it}	0.366	0.001
**Total**	381	1001.304		2.643	100%			
