# Supplementary material for: Genetic analysis and phytochemical profile of soursop (Annona muricata L.) cultivated in family orchards in southeastern Mexico
Source: PLoS One. 2025 May 7;20(5):e0321846. doi: 10.1371/journal.pone.0321846 (PMC12057873; doi:10.1371/journal.pone.0321846)
Supplement: S4 Table — The population structure, evaluated using the Evanno method and based on probability assignments estimated by the STRUCTURE program, suggests that the most probable number of populations is K = 3, represented by three distinct groups. (PDF) [file pone.0321846.s005.pdf]

Three clusters (K = 3)			
**Give of pop**	**1**	**2**	**3**
XA	0.136	0.009	0.856
CZ	0.011	0.010	0.978
CR	0.307	0.467	0.227
HU	0.246	0.012	0.742
CU	0.407	0.007	0.586
CO	0.411	0.015	0.574
PR	0.029	0.771	0.200
NJ	0.015	0.561	0.424
CE	0.141	0.003	0.855
EZ	0.307	0.514	0.179
TE	0.224	0.006	0.770
PA	0.204	0.568	0.228
PI	0.292	0.011	0.697
SA	0.355	0.416	0.229
ST	0.329	0.616	0.056
CH	0.385	0.006	0.609
CA	0.095	0.006	0.898
PZ	0.023	0.012	0.965
